# AI-assisted segmentation-based fusion model integrating deep learning, radiomics, and clinical parameters for identifying coronary heart disease risk in patients with MAFLD

**DOI:** 10.3389/fendo.2026.1908048

**Published:** 2026-08-07

**Authors:** Luying Qi, Shumin Zhang, Bin Wang, Mei Yang, Yuanbin Li, Baiwang Li, Jingnan Xue, Xuemei Li

**Affiliations:** 1 Department of Radiology, First Affiliated Hospital of Huzhou Normal University, Huzhou, Zhejiang, China; 2 Center of Gastrointestinal Endoscopy, The Fourth People’s Hospital of Jinan Affiliated to Shandong Second Medical University, Jinan, Shandong, China; 3 Department of Gastroenterology, The Fourth People’s Hospital of Jinan Affiliated to Shandong Second Medical University, Jinan, Shandong, China

**Keywords:** artificial intelligence, clinical parameters, coronary heart disease, deep learning, metabolic dysfunction-associated fatty liver disease, radiomics, risk prediction

## Abstract

**Background/objectives:**

Metabolic dysfunction-associated fatty liver disease (MAFLD) is associated with an increased risk of coronary heart disease (CHD), which is one of the leading causes of chronic disease-related mortality worldwide. Early identification of CHD risk in patients with MAFLD is essential for risk stratification and timely intervention. Although radiomics and deep learning (DL) have shown promising performance in medical image analysis, their application for CHD risk assessment in patients with MAFLD remains limited. Therefore, this study aimed to develop and validate an AI-assisted segmentation-based deep learning radiomics-clinical (DLRC) model for identifying CHD risk in patients with MAFLD.

**Methods:**

A total of 1,515 patients with MAFLD, including 440 with concomitant CHD, were retrospectively enrolled between January 2023 and December 2025. Patients were randomly divided into a training cohort (n = 1,060) and a test cohort (n = 455). AI-assisted liver segmentation was performed using a deep learning-assisted framework integrated into ITK-SNAP. Radiomics features were extracted using PyRadiomics, and DL features were extracted using a pre-trained DenseNet-121 network. After feature selection using Pearson correlation analysis, minimum redundancy maximum relevance (mRMR), principal component analysis (PCA), and least absolute shrinkage and selection operator (LASSO) regression, radiomics and DL features were integrated to construct a deep learning radiomics (DLR) model. Clinical predictors were identified using multivariable logistic regression. Clinical, radiomics, DLR, and combined DLRC models were developed and evaluated using machine learning algorithms. Model performance was assessed by receiver operating characteristic (ROC) analysis, calibration curves, DeLong tests, and decision curve analysis (DCA).

**Results:**

Multivariable analysis identified older age, hypertension, diabetes mellitus, hyperlipidemia, male sex, and lower BMI as independent predictors of CHD in patients with MAFLD. Eleven radiomics features and fifteen DLR features were selected for model construction. The DLRC model achieved the best predictive performance, with AUCs of 0.917 in the training cohort and 0.878 in the test cohort, outperforming the DLR model (0.895 and 0.854), radiomics model (0.821 and 0.784), clinical model (0.768 and 0.751), respectively. DeLong tests demonstrated significant superiority of the DLRC model over the other models (all P < 0.05). Calibration and DCA analyses further confirmed its excellent calibration and clinical utility.

**Conclusion:**

An AI-assisted segmentation-based fusion model integrating clinical factors, radiomics features, and DL features demonstrated excellent performance for identifying CHD risk in patients with MAFLD. This non-invasive and efficient approach may facilitate early risk stratification and personalized management of MAFLD patients.

## 1 Introduction

In 2020, an international expert consensus statement proposed replacing non-alcoholic fatty liver disease (NAFLD) with metabolic dysfunction-associated fatty liver disease (MAFLD), aiming to eliminate the potential stigmatization associated with the terms “fatty” and “alcoholic” in NAFLD, while adopting affirmative diagnostic criteria instead of the previous exclusionary criteria (1). Currently, approximately one-third (32.4%) of the global adult population is affected by MAFLD, making it the most common chronic liver disease worldwide (2). MAFLD is closely associated with various systemic disorders, not only impairing patients’ health-related quality of life but also increasing healthcare costs and economic burden (3). Multiple studies have shown that MAFLD is significantly associated with an increased risk of cardiovascular disease (CVD) (4–7); however, the definition of cardiovascular outcomes in these studies is relatively broad. Coronary heart disease (CHD) is the most common type of cardiovascular disease and a leading cause of chronic disease-related mortality worldwide (8). In recent investigations, MAFLD has been demonstrated to be associated with an increased risk of CHD (9). The specific mechanisms underlying the association between MAFLD and CHD remain unclear, with genetic susceptibility, insulin resistance (IR), dyslipidemia, oxidative stress, inflammation, and endothelial dysfunction considered key links mediating the increased CHD risk in NAFLD (10). Therefore, early detection of MAFLD patients and timely intervention are of paramount importance.

Artificial intelligence (AI) has achieved significant progress in the fields of disease diagnosis and medical image analysis (11, 12). Radiomics employs high-throughput automated extraction algorithms to evaluate features such as geometric shape and texture that are imperceptible to the human visual system, transforming traditional images that rely on visual interpretation into quantitative features (13). Deep learning (DL), as an important branch of AI, is particularly adept at identifying complex features in radiological images. Through multi-layer neural network (NN) models and utilizing large-scale data, DL achieves efficient learning and feature extraction, thereby improving the accuracy of disease diagnosis and assessment (11, 14). Currently, radiomics and DL have been extensively studied in malignant tumors, including lung cancer, colorectal cancer, gastric cancer, and breast cancer (15–18); however, their application in chronic diseases remains relatively limited.

To date, no study has utilized AI-assisted segmentation-based DL radiomics to predict CHD risk in patients with MAFLD. In the present study, we integrated DL features, radiomics features, and clinical variables into a final predictive model and evaluated its diagnostic performance for identifying CHD in patients with MAFLD.

## 2 Methods

This retrospective study was designed to develop and validate an AI-assisted segmentation-based DLRC model for CHD risk identification in patients with MAFLD. The overall workflow consisted of five major steps: patient selection, liver CT preprocessing and AI-assisted segmentation, extraction of radiomics and deep learning features, integration with clinical parameters, and model evaluation.

### 2.1 Patient selection and clinical data

**Figure 1:**
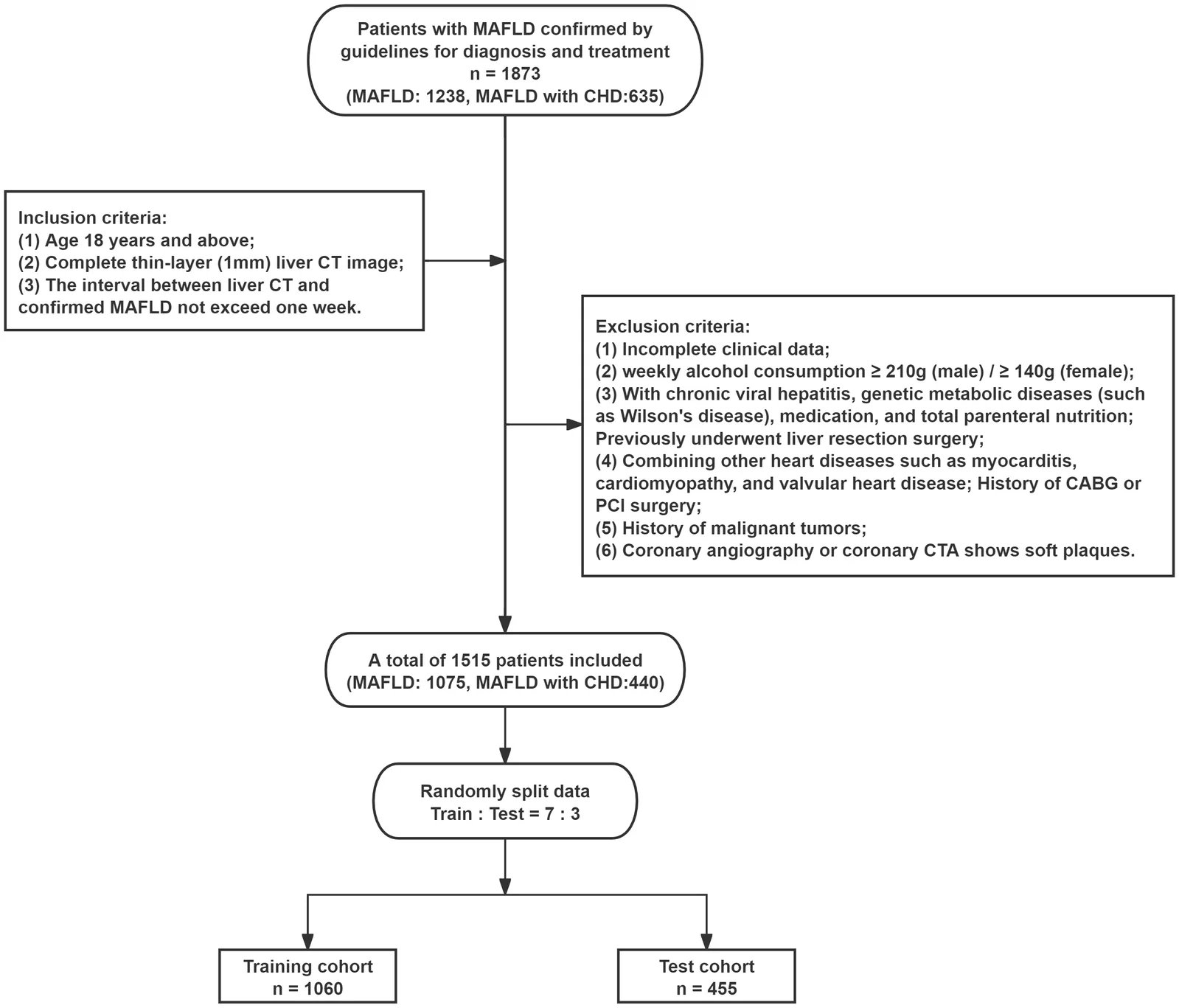
Flowchart of patient selection.

This retrospective study was approved by the Ethics Committee of First Affiliated Hospital of Huzhou Normal University (Approval Number: 2025KYLL099-01), which waived the requirement for informed consent. Clinical data and CT images were collected from 1,515 eligible patients (1,075: MAFLD without CHD and 440: MAFLD with CHD) between January 2023 and December 2025. Inclusion criteria were: (1) age ≥ 18 years; (2) diagnosis of MAFLD according to the Primary Care Guidelines for the Diagnosis and Management of Metabolic Dysfunction-Associated Fatty Liver Disease (2025) (19); (3) availability of complete thin-slice (1-mm) liver CT images; and (4) liver CT performed within one week of MAFLD diagnosis. Exclusion criteria were: (1) incomplete clinical data; (2) alcohol consumption ≥ 210 g/week in male patients or ≥ 140 g/week in female patients; (3) presence of specific factors leading to fatty liver, including chronic viral hepatitis, hereditary metabolic diseases (e.g., Wilson’s disease), drug-induced liver disease, total parenteral nutrition, or a history of liver resection; (4) other cardiac diseases (e.g., myocarditis, cardiomyopathy, valvular heart disease) or history of CABG/PCI; (5) history of malignancy; or (6) soft plaques on coronary angiography/CTA. The 1,515 patients were randomly divided into a training cohort (n = 1,060) and a test cohort (n = 455). **Figure 1** shows the patient selection flowchart.

Clinical information included sex, age, body mass index (BMI), hypertension, diabetes mellitus, hyperlipidemia, smoking status, and hepatic insufficiency status. Laboratory indicators included white blood cell count (WBC), neutrophil count (NEU), absolute lymphocyte count (LYM), absolute monocyte count (MONO), hemoglobin (Hb), platelet count (PLT), albumin (ALB), glucose (Glu), triglycerides (TG), total cholesterol (TC), high-density lipoprotein cholesterol (HDL-C), low-density lipoprotein cholesterol (LDL-C), aspartate aminotransferase (AST), alanine aminotransferase (ALT), total bilirubin (TBIL), creatinine (Cr), blood urea nitrogen (BUN), uric acid (UA), and the albumin-to-globulin ratio (A/G).

### 2.2 Image acquisition and preprocessing

Patients were instructed to hold their breath after calm inspiration, and non-contrast computed tomography (CT) of the liver was performed. Each patient was examined using one of the following scanners: Somatom Definition AS (Siemens Healthineers), Somatom Perspective (Siemens Healthineers), or GE Optima 540 (GE Healthcare). Detailed scanning parameters are provided in **Supplementary**. All images were standardized through a three-step preprocessing procedure. First, all images were converted from DCM format to nii.gz format. Second, images were resampled to a voxel size of 1 × 1 × 1 mm³. Finally, a uniform window width of 400 Hounsfield units (HU) and a window level of 70 HU were applied to all images.

### 2.3 AI-assisted liver image segmentation

AI-assisted liver segmentation was performed using the deep learning server (DLS) within ITK-SNAP (version 4.4), a Python-based extension that integrates nnInteractive’s deep learning interactive segmentation functions into the ITK-SNAP platform. After simple annotation with a brush tool, the system automatically generated an accurate 3D liver segmentation within seconds, significantly reducing manual workload and improving efficiency. All imaging data were anonymized before evaluation, and any patient identifiers were removed. All generated liver masks were visually inspected to ensure anatomical accuracy, and necessary manual corrections were performed by a senior radiologist when discrepancies were identified, particularly in regions with unclear liver boundaries, adjacent organs, or heterogeneous liver parenchyma. The entire liver volume was then extracted as the region of interest (ROI) and used as input for the subsequent 3D deep learning model.

### 2.4 Feature extraction and model development

Radiomics features were extracted using the PyRadiomics package within the OnekeyAI platform. All features were normalized using Z-score normalization to eliminate discrepancies caused by differences in units, scales, and imaging equipment across features. To reduce multicollinearity, features with a Pearson correlation coefficient > 0.9 were iteratively excluded. The minimum redundancy maximum relevance (mRMR) method was then applied to remove irrelevant and redundant features. Subsequently, LASSO regression was employed to reduce the dimensionality of the initial features, and weights were assigned to the selected features. Features with higher weights were retained and used as inputs for machine learning classifiers in model development. To minimize overfitting, feature selection procedures were performed exclusively within the training cohort, and five-fold cross-validation was used to determine the optimal LASSO penalty parameter before final model construction.

DL features were extracted using the ImageNet-pretrained DenseNet-121 model via transfer learning. DenseNet-121 is a classic convolutional neural network (CNN) architecture, and was employed to extract DL features in this study. The extracted DL features were compressed using principal component analysis (PCA) and subsequently normalized using Z-score standardization. LASSO regression was then applied to filter features with non-zero coefficients, thereby reducing dimensionality and selecting the compressed DL features. Radiomics features and compressed DL features were integrated to develop deep learning radiomics (DLR) features. After LASSO-based feature selection, the resulting DLR features were used as inputs for model development.

**Figure 2:**
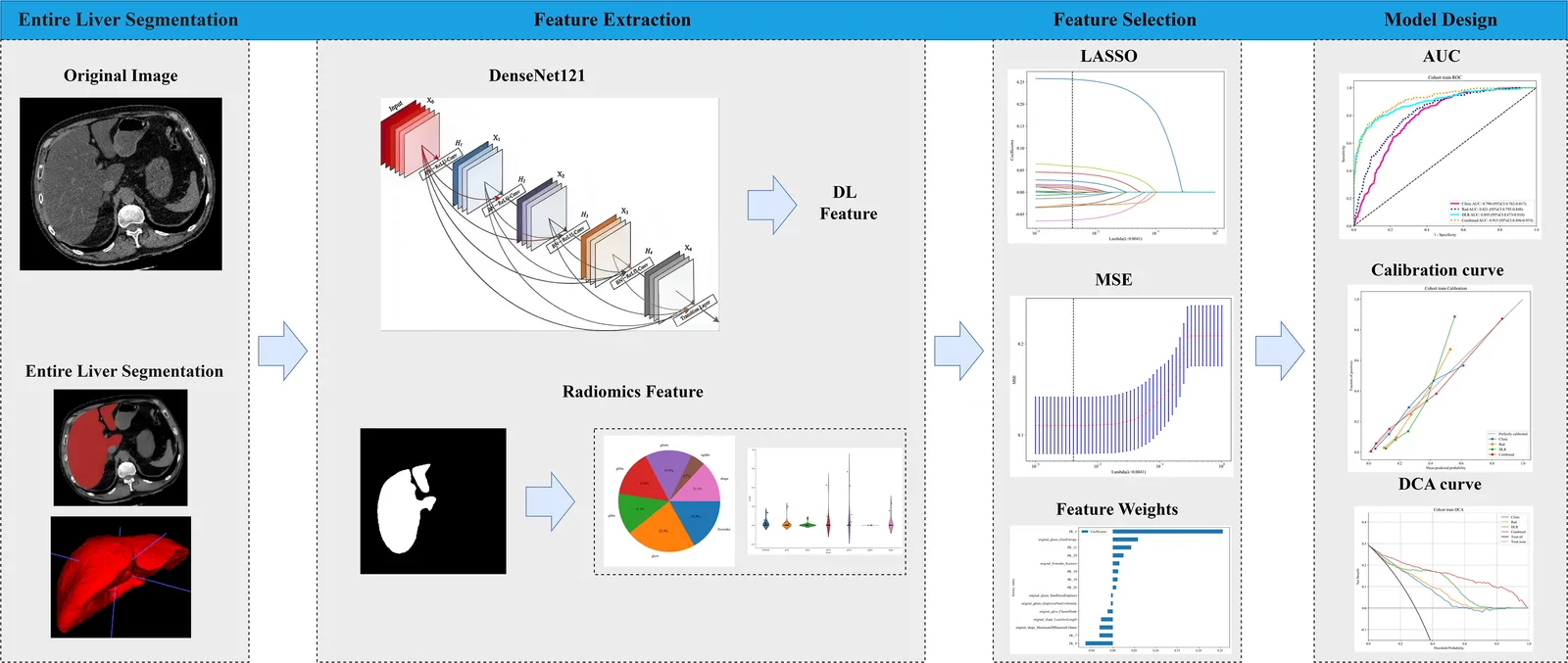
Flowchart of the study design.

For clinical features, univariable and multivariable logistic regression analyses were performed. Variables with a P-value < 0.05 in univariable analysis were entered into the multivariable analysis to identify statistically significant features for constructing the clinical model. The clinical features, radiomics features, and DLR features were separately fed into eight machine learning classifiers, including logistic regression (LR), support vector machine (SVM), K-nearest neighbors (KNN), random forest (RF), ExtraTrees, gradient boosting (XGBoost), light gradient boosting machine (LightGBM), and multilayer perceptron (MLP), to develop models. The optimal models based on clinical, radiomics, and DLR features were selected respectively. The clinical model and the DLR model were subsequently integrated to create the final predictive model in this study, which was termed the DLRC model (deep learning-radiomics-clinical model). Model performance was evaluated using the following metrics: accuracy, area under the curve (AUC) with 95% confidence interval (CI), sensitivity, specificity, positive predictive value (PPV), negative predictive value (NPV), precision, recall, and F1-score. Calibration curves were used to assess the calibration performance of the combined model in both the training and test datasets, and the Hosmer–Lemeshow test was applied to evaluate goodness-of-fit. Decision curve analysis (DCA) was performed to assess the net benefit of the model across different probability thresholds. The study design workflow is illustrated in **Figure 2**.

### 2.5 Statistical analysis

Statistical analysis of clinical variables was performed using SPSS (version 26.0). Continuous variables with a normal distribution were compared using the independent samples t-test, while those with a non-normal distribution were compared using the Mann–Whitney U test. Categorical variables are presented as n (%) and were compared using the chi-square test. Baseline comparisons between CHD and non-CHD groups were performed using independent samples t-tests, Mann–Whitney U test or chi-square tests. Univariable and multivariable binary logistic regression analyses were performed to identify independent clinical risk factors associated with CHD in patients with MAFLD. Variables with statistical significance in univariable analyses were subsequently entered into multivariable logistic regression analysis. A P-value < 0.05 was considered statistically significant. Feature extraction and modeling analyses for radiomics, deep learning, and clinical variables were all performed using the Python environment on the OnekeyAI platform (version 4.9.1).

Based on the above workflow, clinical characteristics, imaging features, DLR features, and integrated predictive models were subsequently analyzed and compared between patients with and without CHD.

## 3 Results

### 3.1 Clinical characteristics and univariate and multivariate analyses

As of December 2025, a total of 1,515 eligible patients with MAFLD were enrolled and randomly divided into a training cohort (n = 1,060; 749 with MAFLD without CHD and 311 with MAFLD with CHD) and a test cohort (n = 455; 326 with MAFLD without CHD and 129 with MAFLD with CHD) at a ratio of 7:3. The study population comprised 903 males and 612 females. In both the training and test cohorts, statistically significant differences were observed between the non-CHD and CHD groups in terms of age, ALB, TC, LDL-C, Cr, BUN, and hypertension (all P < 0.05). Additionally, in the training cohort, significant differences were also noted between the two groups in WBC, NEU, MONO, Hb, HDL-C, ALT, A/G, diabetes, and hepatic insufficiency (all P < 0.05). Detailed information is presented in **Table 1**.

**Table 1:** Baseline characteristics of MAFLD patients with and without CHD in the training and test cohorts.

Clinical factors	Training cohort (n=1060)		P value	Test cohort (n=455)		P value
	MAFLD without CHD (n=749)	MAFLD with CHD (n=311)		MAFLD without CHD (n=326)	MAFLD with CHD (n=129)	
Age (years)	51.54 ± 15.40	63.74 ± 10.66	<0.001	52.84 ± 15.58	63.62 ± 11.18	<0.001
BMI (kg/m²)	27.04 ± 4.44	26.57 ± 3.64	0.292	26.83 ± 3.95	26.82 ± 3.26	0.564
WBC (×10^9^/L)	8.14 ± 3.68	6.61 ± 2.03	<0.001	7.59 ± 3.36	6.91 ± 2.61	0.124
NEU (×10^9^/L)	5.92 ± 3.61	4.37 ± 1.79	<0.001	5.42 ± 3.21	5.02 ± 4.87	0.128
LYM (×10^9^/L)	1.63 ± 0.83	1.68 ± 0.64	0.055	1.63 ± 0.80	1.72 ± 0.66	0.045
MONO (×10^9^/L)	0.46 ± 0.24	0.39 ± 0.17	<0.001	0.42 ± 0.22	0.40 ± 0.15	0.997
Hb (g/L)	140.99 ± 20.75	143.58 ± 17.11	0.038	141.08 ± 19.32	142.90 ± 18.09	0.223
PLT (×10^9^/L)	219.16 ± 76.23	209.80 ± 58.36	0.099	217.03 ± 70.18	205.33 ± 59.90	0.110
ALB (g/L)	41.73 ± 5.00	43.26 ± 3.72	<0.001	42.30 ± 4.34	43.34 ± 3.46	0.016
Glu (mmol/L)	7.63 ± 3.68	7.61 ± 3.42	0.529	7.56 ± 3.48	7.28 ± 2.92	0.853
TG (mmol/L)	3.71 ± 7.05	2.29 ± 1.58	0.758	3.84 ± 7.24	2.43 ± 1.28	0.597
TC (mmol/L)	5.29 ± 1.95	4.68 ± 1.36	<0.001	5.42 ± 2.14	4.63 ± 1.13	<0.001
HDL-C (mmol/L)	74.95 ± 33.59	81.08 ± 29.96	0.010	78.43 ± 31.95	76.03 ± 25.83	0.455
LDL-C (mmol/L)	2.73 ± 0.93	2.52 ± 0.97	0.002	2.76 ± 0.92	2.52 ± 0.89	0.026
AST (U/L)	36.19 ± 49.81	31.06 ± 25.06	0.446	40.36 ± 53.49	30.07 ± 15.73	0.131
ALT (U/L)	47.35 ± 63.41	37.03 ± 28.87	0.015	50.05 ± 70.29	37.09 ± 25.16	0.073
TBIL (μmol/L)	17.29 ± 12.58	16.09 ± 10.30	0.580	17.50 ± 13.31	15.94 ± 8.05	0.474
Cr (μmol/L)	77.92 ± 53.42	79.24 ± 27.44	<0.001	76.26 ± 26.95	86.12 ± 55.52	0.012
BUN (mmol/L)	5.46 ± 2.71	5.99 ± 2.23	<0.001	5.49 ± 2.10	6.24 ± 2.03	<0.001
UA (μmol/L)	380.01 ± 135.07	385.56 ± 104.78	0.169	380.26 ± 112.38	383.71 ± 105.91	0.355
A/G	1.48 ± 0.27	1.53 ± 0.24	0.004	1.48 ± 0.27	1.51 ± 0.22	0.295
Sex			0.712			0.061
Female	295(39.39)	118(37.94)		152(46.63)	47(36.43)	
Male	454(60.61)	193(62.06)		174(53.37)	82(63.57)	
Hypertension			<0.001			<0.001
No	401(53.54)	69(22.19)		159(48.77)	25(19.38)	
Yes	348(46.46)	242(77.81)		167(51.23)	104(80.62)	
Diabetes			<0.001			0.573
No	522(69.69)	180(57.88)		223(68.40)	84(65.12)	
Yes	227(30.31)	131(42.12)		103(31.60)	45(34.88)	
Hyperlipidemia			0.920			0.954
No	579(77.30)	242(77.81)		256(78.53)	101(78.29)	
Yes	170(22.70)	69(22.19)		70(21.47)	28(21.71)	
Smoking			0.457			0.943
No	590(78.77)	252(81.03)		255(78.22)	102(79.07)	
Yes	159(21.23)	59(18.97)		71(21.78)	27(20.93)	
Hepatic Insufficiency			0.022			0.213
No	624(83.31)	277(89.07)		274(84.05)	115(89.15)	
Yes	125(16.69)	34(10.93)		52(15.95)	14(10.85)	

To identify independent clinical predictors associated with CHD, univariable binary logistic regression analyses were first performed in the training cohort. Variables were then entered into multivariable logistic regression analysis when P values < 0.05. After adjustment for confounders, hyperlipidemia, male sex, diabetes, hypertension, lower BMI, and older age remained independently associated with CHD (all P < 0.05). Variance inflation factor (VIF) values were < 5 for all variables, indicating no significant collinearity. The OR (95% CI) were 1.949 (1.376–2.759), 1.600 (1.124–2.277), 1.447 (1.064–1.970), 2.541 (1.896–3.408), 0.932 (0.900–0.965), and 1.040 (1.029–1.051), respectively. Hyperlipidemia, male sex, diabetes, hypertension, lower BMI, and older age are associated with higher CHD risk in MAFLD patients, with hypertension being the strongest predictor. These variables were used to develop the clinical model (**Table 2**).

**Table 2:** Univariable and multivariable logistic regression analyses of clinical factors associated with CHD.

Clinical factors	Univariable analysis		Multivariable analysis	
	OR (95% CI)	P value	OR (95% CI)	P value
Age (years)	0.989 (0.987–0.991)	<0.001	1.040 (1.029–1.051)	<0.001
BMI (kg/m²)	0.968 (0.964–0.971)	<0.001	0.932 (0.900–0.965)	0.001
WBC (×10^9^/L)	0.881 (0.868–0.895)	<0.001	1.065 (0.749–1.514)	0.769
NEU (×10^9^/L)	0.835 (0.817–0.853)	<0.001	0.784 (0.549–1.120)	0.261
LYM (×10^9^/L)	0.638 (0.598–0.680)	<0.001	1.033 (0.692–1.542)	0.894
MONO (×10^9^/L)	0.133 (0.103–0.171)	<0.001	0.319 (0.122–0.838)	0.052
Hb (g/L)	0.994 (0.993–0.995)	<0.001	1.006 (0.997–1.015)	0.279
PLT (×10^9^/L)	0.996 (0.996–0.997)	<0.001	1.001 (0.999–1.004)	0.295
ALB (g/L)	0.980 (0.978–0.983)	<0.001	0.999 (0.960–1.039)	0.952
Glu (mmol/L)	0.905 (0.892–0.918)	<0.001	0.990 (0.946–1.037)	0.715
TG (mmol/L)	0.763 (0.731–0.796)	<0.001	0.958 (0.861–1.067)	0.518
TC (mmol/L)	0.834 (0.815–0.852)	<0.001	0.775 (0.593–1.012)	0.116
HDL-C (mmol/L)	0.991 (0.990–0.992)	<0.001	1.005 (1.000–1.011)	0.127
LDL-C (mmol/L)	0.722 (0.694–0.753)	<0.001	0.863 (0.629–1.184)	0.442
AST (U/L)	0.978 (0.975–0.981)	<0.001	1.000 (0.990–1.009)	0.924
ALT (U/L)	0.982 (0.979–0.985)	<0.001	0.998 (0.990–1.005)	0.615
TBIL (μmol/L)	0.954 (0.948–0.961)	<0.001	0.985 (0.969–1.001)	0.119
Cr (μmol/L)	0.990 (0.989–0.991)	<0.001	0.995 (0.988–1.001)	0.142
BUN (mmol/L)	0.883 (0.867–0.900)	<0.001	1.003 (0.932–1.078)	0.955
UA (μmol/L)	0.998 (0.998–0.998)	<0.001	1.001 (1.000–1.003)	0.165
A/G	0.578 (0.537–0.622)	<0.001	0.671 (0.362–1.241)	0.286
Sex	0.425 (0.369–0.490)	<0.001	1.600 (1.124–2.277)	0.029
Hypertension	0.695 (0.606–0.798)	<0.001	2.541 (1.896–3.408)	<0.001
Diabetes	0.577 (0.482–0.691)	<0.001	1.447 (1.064–1.970)	0.048
Hyperlipidemia	0.406 (0.321–0.513)	<0.001	1.949 (1.376–2.759)	0.002
Smoking	0.371 (0.289–0.477)	<0.001	1.101 (0.773–1.570)	0.655
Hepatic Insufficiency	0.272 (0.198–0.374)	<0.001	0.827 (0.532–1.287)	0.479

### 3.2 Feature selection and model development

**Figure 3:**
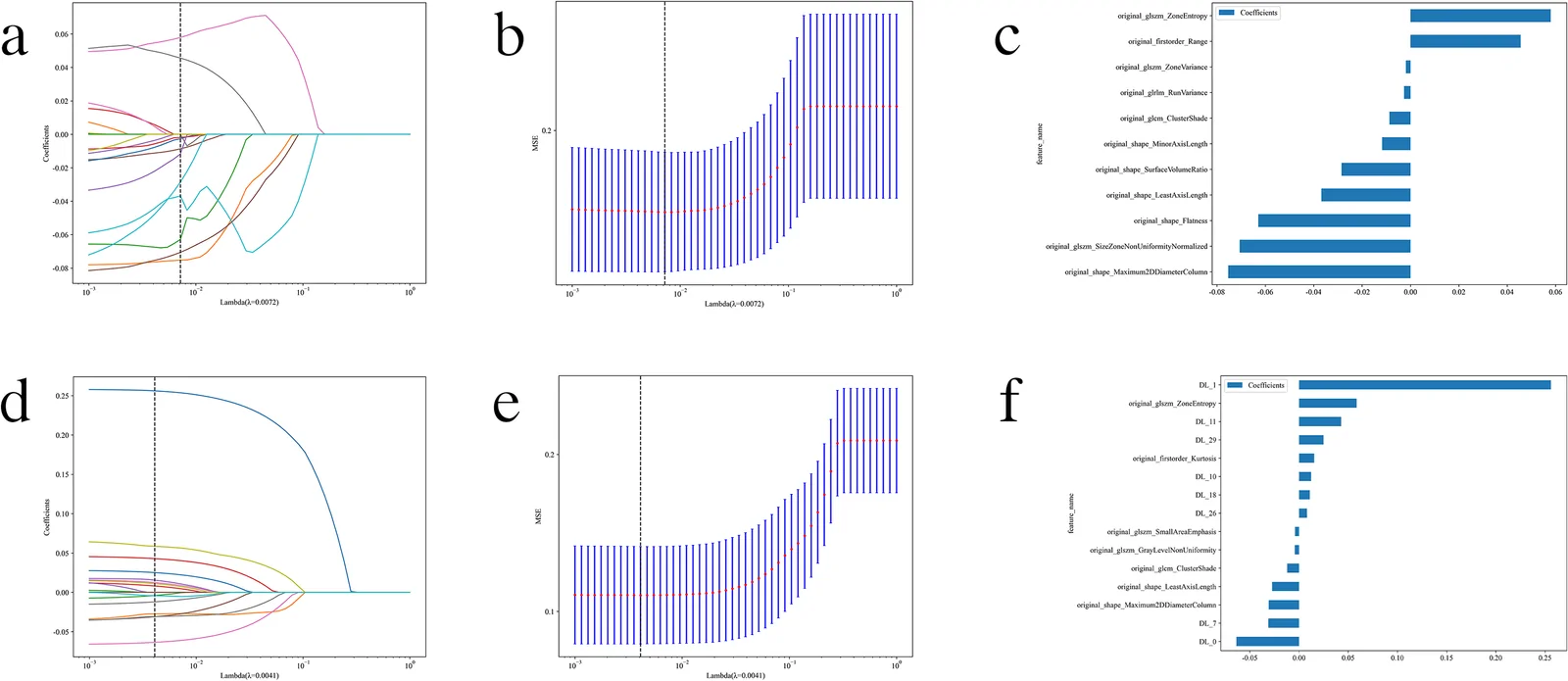
**(a, d)** LASSO coefficient paths for radiomics and DLR features; **(b, e)** MSE curves for radionics and DLR features; **(c, f)** Feature weights for radionics and DLR features.

A total of 107 radiomics features were extracted. After Z-score normalization, Pearson correlation, mRMR, and LASSO regression with five-fold cross-validation, the optimal λ value was determined as 0.0072, yielding 11 radiomics features with non-zero coefficients. These features were used to construct the radiomics model. Subsequently, 31 compressed DL features were integrated with the radiomics features. Following Pearson correlation, LASSO regression, and five-fold cross-validation (λ = 0.0041), 15 DLR features with non-zero coefficients were selected to construct the DLR model. The Radscore and DLRscore are provided in **Supplementary**. Visualization of LASSO coefficient paths, MSE curves, and feature weights for both radiomics and DLR features is presented in **Figure 3**.

**Figure 4:**
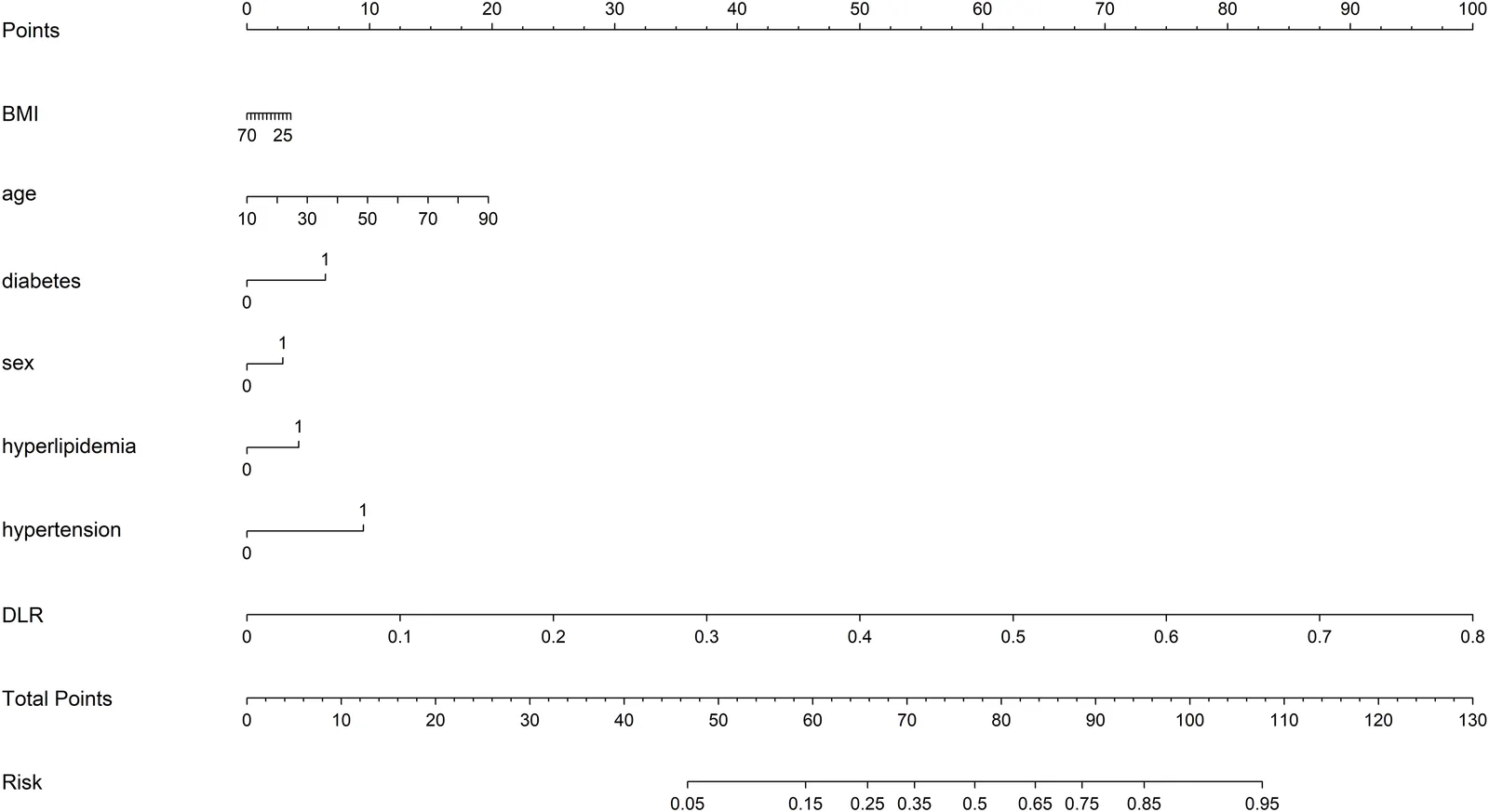
Nomogram of the DLRC model.

The DLR features were combined with the selected clinical features to construct a fusion model, which we termed the DLRC model. The nomogram of this model, which can predict the probability of CHD based on multiple indicators, is shown in **Figure 4**. Our nomogram demonstrated that the DLR score was the most important predictor, followed by age.

**Figure 5:**
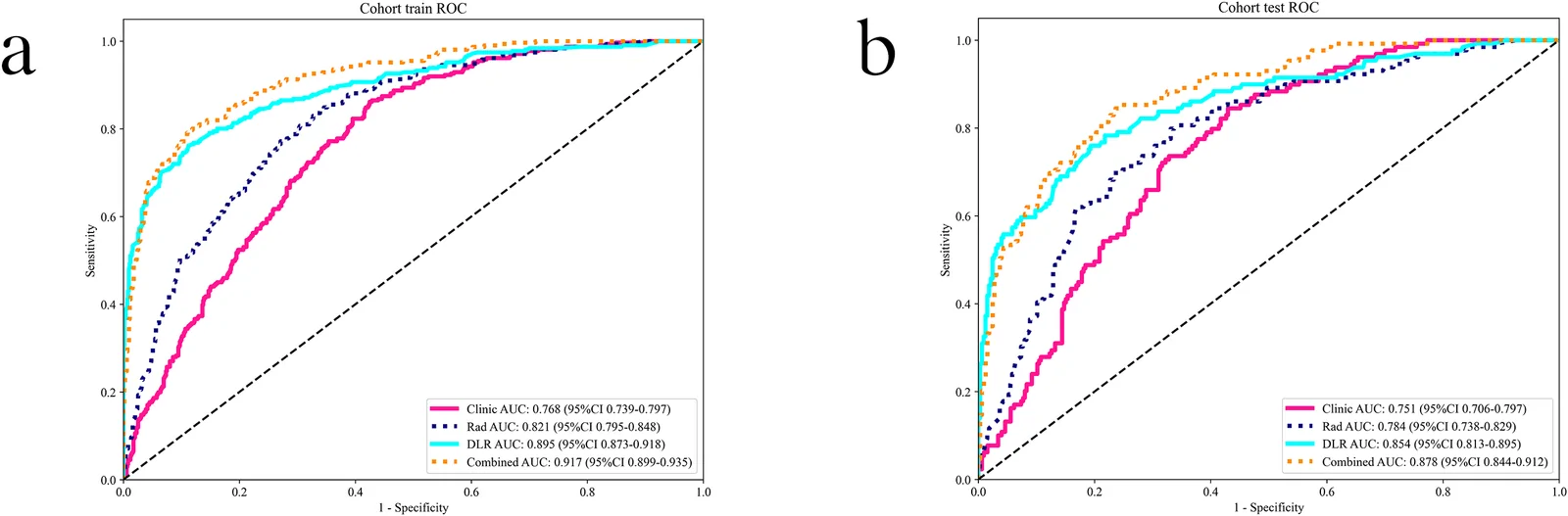
ROC curves of different models **(a)** training cohort; **(b)** testing cohort.

**Figure 6:**
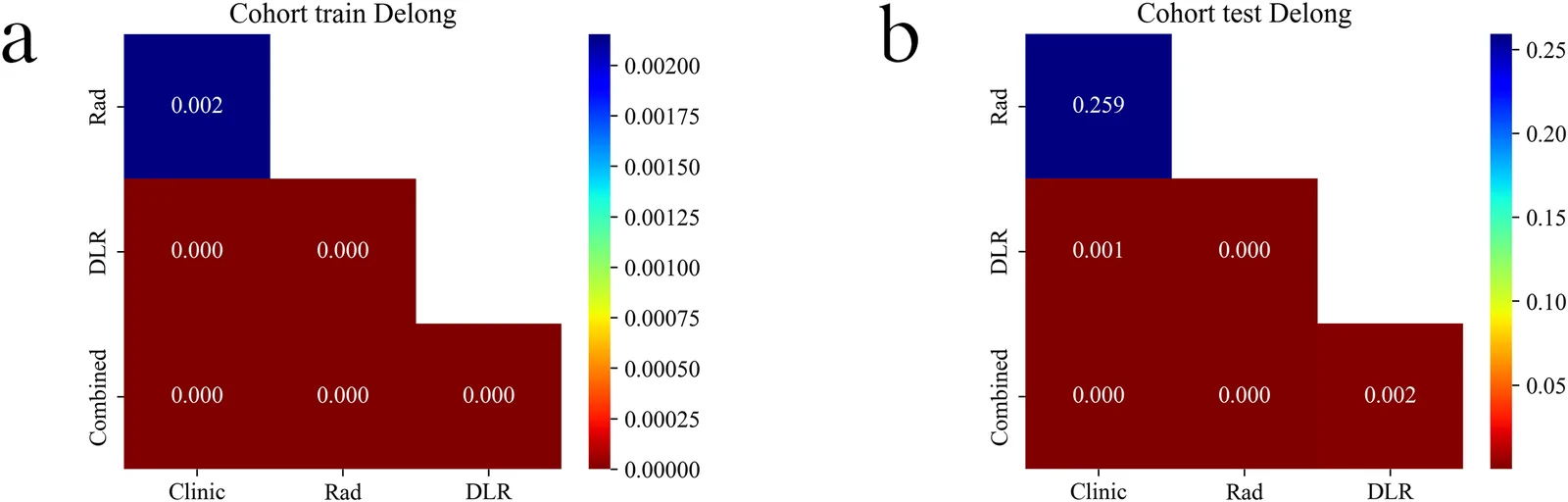
DeLong test results for comparing the AUCs of different models **(a)** training cohort; **(b)** testing cohort.

**Table 3** summarizes the performance of the four models. In the training cohort, the fusion model achieved the highest AUC (0.917), followed by the DLR model (0.895), radiomics model (0.821), and clinical model (0.768). Similar trends were observed in the test cohort, with AUCs of 0.878, 0.854, 0.784, and 0.751, respectively. The fusion model consistently outperformed the other models in both cohorts. The receiver operating characteristic (ROC) curves are displayed in **Figure 5**. DeLong tests confirmed that the combined model significantly outperformed the clinical, radiomics, and DLR models in both cohorts (all P < 0.05), as shown in **Figure 6**.

**Table 3:** Performance comparison of the four models in the training and test cohorts.

Signature	Accuracy	AUC(95% CI)	Sensitivity	Specificity	PPV	NPV	Precision	Recall	F1	Threshold	Cohort
Clinic	0.659	0.768 (0.739–0.797)	0.862	0.575	0.457	0.909	0.457	0.862	0.598	0.222	train
Rad	0.717	0.821 (0.795–0.848)	0.836	0.668	0.511	0.907	0.511	0.836	0.634	0.283	train
DLR	0.851	0.895 (0.873–0.918)	0.762	0.888	0.738	0.900	0.738	0.762	0.750	0.370	train
Combined	0.861	0.917 (0.899–0.935)	0.794	0.889	0.748	0.912	0.748	0.794	0.771	0.381	train
Clinic	0.648	0.751 (0.706–0.797)	0.845	0.571	0.438	0.903	0.438	0.845	0.577	0.240	test
Rad	0.705	0.784 (0.738–0.830)	0.798	0.669	0.488	0.893	0.488	0.798	0.606	0.284	test
DLR	0.793	0.854 (0.813–0.895)	0.760	0.807	0.609	0.895	0.609	0.760	0.676	0.323	test
Combined	0.787	0.878 (0.844–0.912)	0.845	0.764	0.586	0.926	0.586	0.845	0.692	0.202	test

### 3.3 Comparison of clinical utility among different models

**Figure 7:**
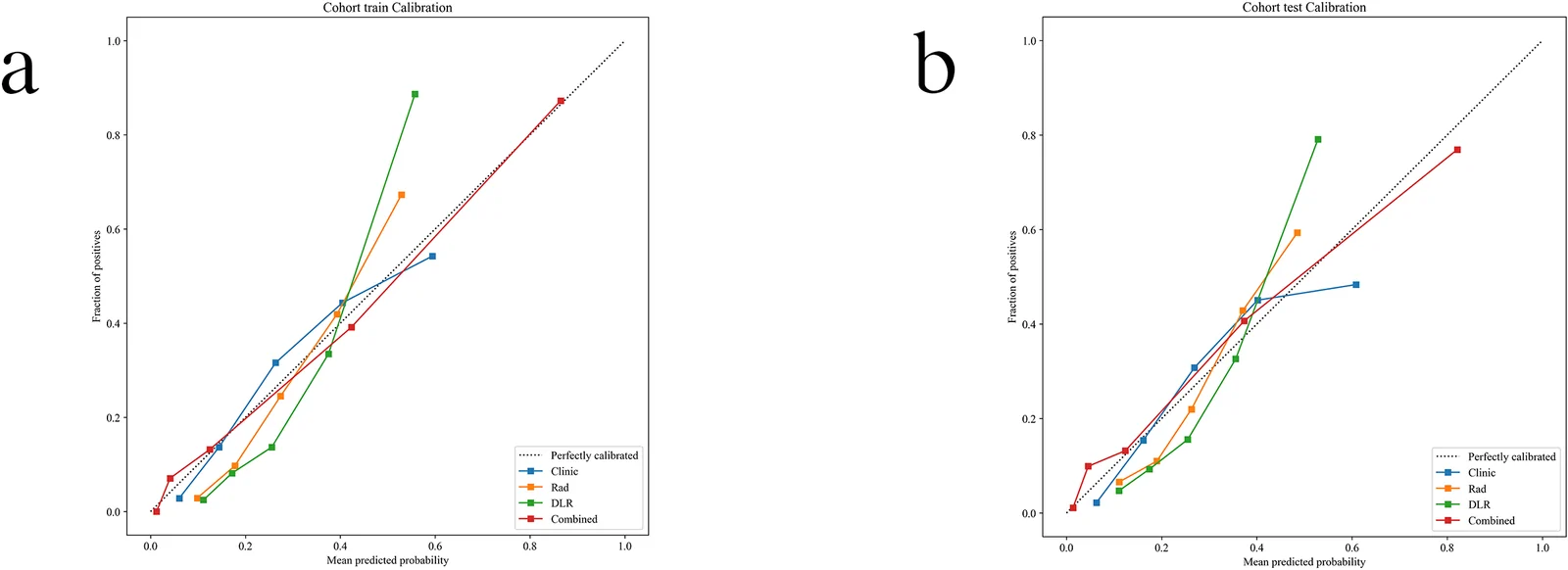
Calibration curves of different models **(a)** training cohort; **(b)** testing cohort.

**Figure 8:**
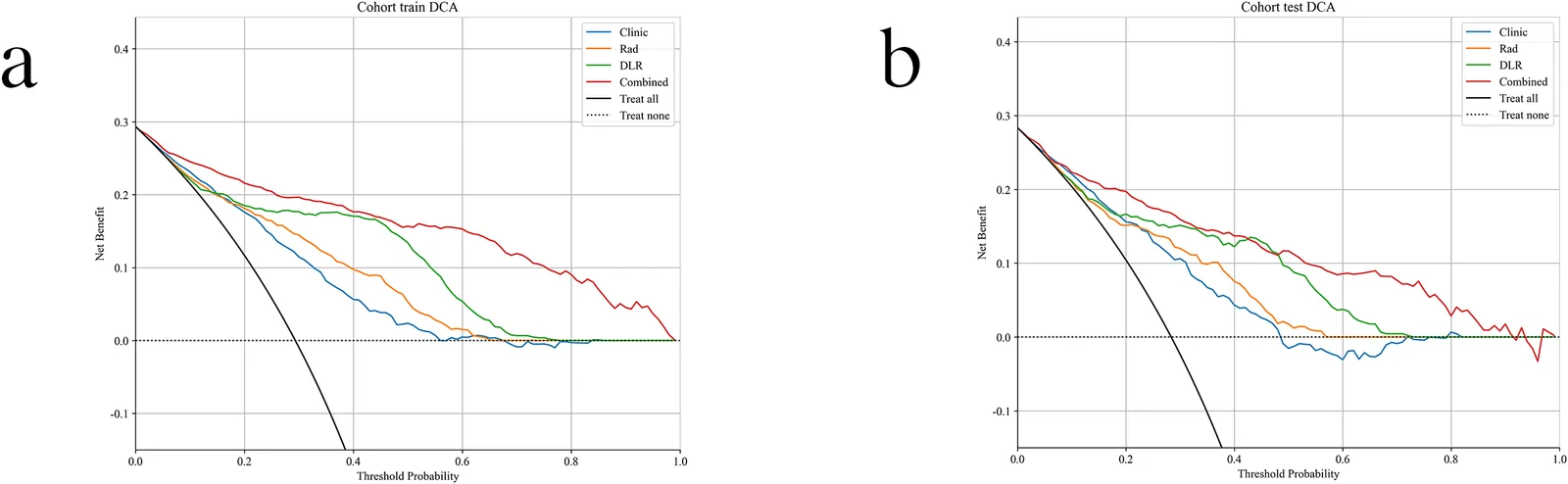
Decision curve analysis of different models **(a)** training cohort; **(b)** testing cohort.

Calibration curves are shown in **Figure 7**. The results demonstrated that the actual reference lines of all models were in good agreement with the ideal reference lines, with the fusion model exhibiting the best calibration performance. DCA was further performed to evaluate the clinical utility of the models. The results showed that the fusion model achieved a wider range of net benefit across threshold probabilities compared to the other models. Moreover, within most threshold probability ranges, the fusion model yielded higher net benefits than the DLR, radiomics, and clinical models (**Figure 8**).

### 3.4 Subgroup analysis

**Figure 9:**
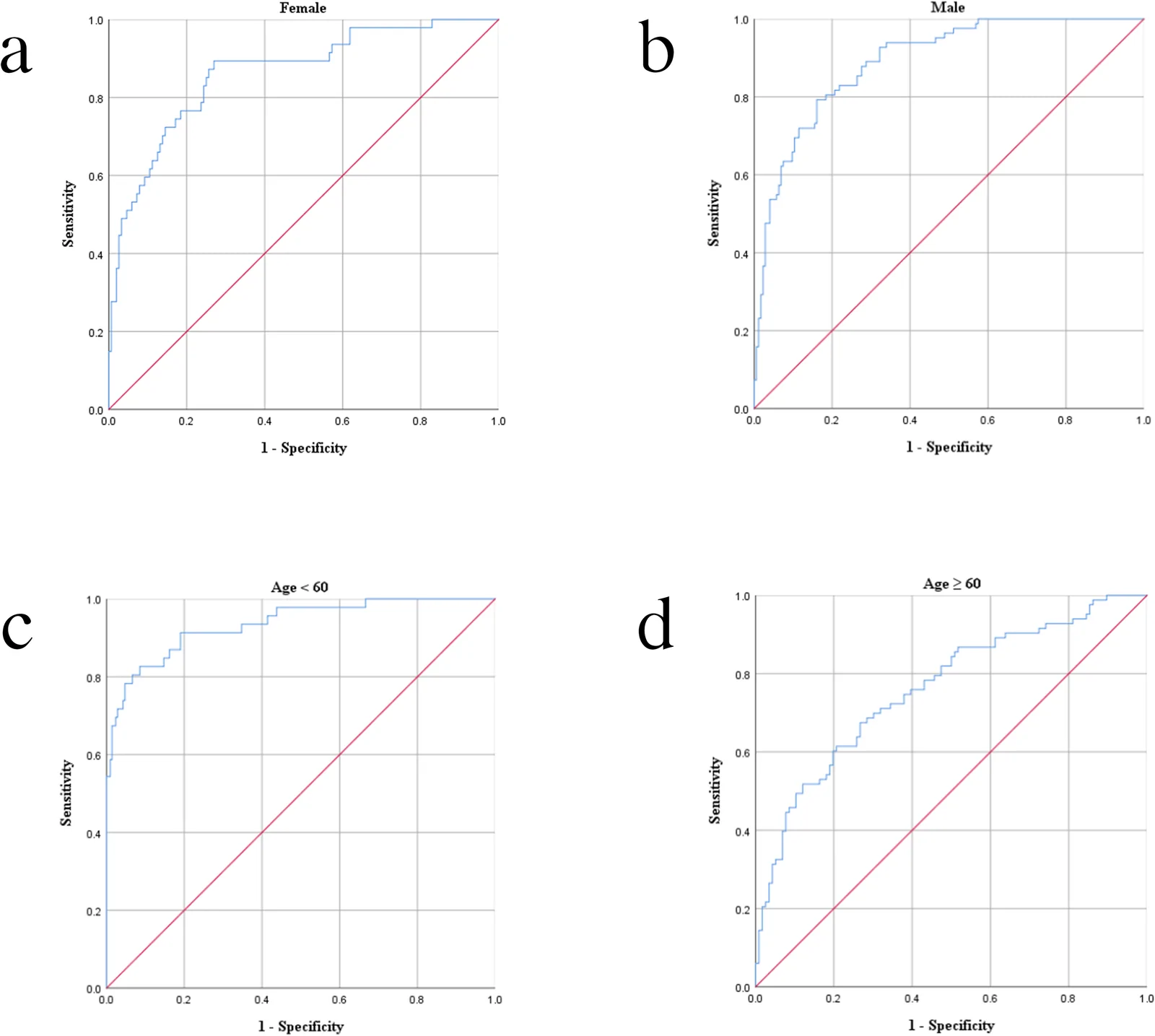
ROC curves of the combined model in different sex and age subgroups **(a)** females; **(b)** males; **(c)** patients aged <60 years; **(d)** patients aged ≥60 years.

Further subgroup analyses were performed in the test cohort to evaluate the robustness of the combined model. The same classification threshold was applied across all subgroups to ensure objective comparison and avoid subgroup-specific optimization, which may introduce overfitting. The results demonstrated that the combined model maintained good discriminatory ability across different sex and age subgroups. The details are shown in **Figure 9** and **Table 4**.

**Table 4:** Subgroup analysis of the combined model performance in the test cohort.

Subgroup		n	AUC	95%CI	Sensitivity	Specificity
Sex	Female	199	0.863	0.801-0.925	0.872	0.743
	Male	256	0.891	0.852-0.931	0.829	0.782
Age (years)	Age < 60	256	0.935	0.894-0.977	0.826	0.914
	Age ≥ 60	199	0.760	0.692-0.828	0.855	0.491

In the sex subgroup analysis, the combined model achieved an AUC of 0.863 (95% CI: 0.801–0.925) in female patients and 0.891 (95% CI: 0.852–0.931) in male patients, indicating comparable predictive performance between the two groups. The sensitivity and specificity were 0.872 and 0.743 in females and 0.829 and 0.782 in males, respectively. In the age subgroup analysis, the combined model showed superior performance in patients aged <60 years, with an AUC of 0.935 (95% CI: 0.894–0.977), sensitivity of 0.826, and specificity of 0.914. The model demonstrated moderate discrimination in patients aged ≥60 years, with an AUC of 0.760 (95% CI: 0.692–0.828). Although the sensitivity remained high (0.855), the specificity decreased to 0.491 in this subgroup.

Collectively, these findings demonstrate that integrating DLR features with clinical variables improves CHD risk prediction in MAFLD patients, providing a foundation for further interpretation of the clinical implications and potential applications of the proposed model.

## 4 Discussion

To our knowledge, this study represents one of the first attempts to develop a deep learning radiomics nomogram based on AI-assisted segmentation for CHD risk stratification in patients with MAFLD. Previous studies have developed clinical prediction models and nomograms to estimate CHD risk in MAFLD populations (8), but imaging-based deep learning radiomics approaches remain insufficiently explored. Recent studies have demonstrated that integrating deep learning features, radiomics signatures, and clinical variables can significantly improve predictive performance compared with single-modality models (11, 20–22). Therefore, by integrating radiomics features and DL features extracted from non-contrast liver CT with clinical risk factors, we established a combined DLRC model that exhibited excellent discrimination, calibration, and clinical utility in predicting the risk of CHD in patients with MAFLD. The combined model achieved AUCs of 0.917 and 0.878 in the training and test cohorts, respectively, outperforming the clinical, radiomics, and DLR models individually. These findings indicate that quantitative liver imaging features combined with clinical characteristics may offer valuable information for predicting CHD risk in MAFLD patients.

MAFLD is increasingly recognized as a multisystem metabolic disorder rather than a liver-confined disease (1, 23). Accumulating evidence shows that cardiovascular disease is the leading cause of mortality in MAFLD patients (5, 24, 25). In our study, multivariable logistic regression identified advanced age, hypertension, diabetes, hyperlipidemia, and male sex as independent predictors of CHD in MAFLD patients, consistent with previous epidemiological studies (26–29), indicating that traditional cardiometabolic risk factors play key roles in coronary atherosclerosis development. Among these, hypertension had the strongest predictive effect (OR = 2.541), underscoring the importance of blood pressure control. The associations of diabetes and hyperlipidemia with CHD may reflect shared pathophysiological mechanisms, including IR, dyslipidemia, chronic inflammation, oxidative stress, and endothelial dysfunction (4, 30–32). Interestingly, lower BMI was associated with increased CHD risk, which may be related to age-related muscle loss, body composition changes, or the “obesity paradox” (33–36); however, this finding requires further investigation and should be interpreted cautiously.

Radiomics has emerged as a promising tool for extracting high-dimensional quantitative data from medical images. In our study, the radiomics model achieved AUCs of 0.821 and 0.784 in the training and test cohorts, suggesting that liver CT images harbor imaging features associated with CHD risk. Prior studies have confirmed that hepatic steatosis is closely linked to systemic metabolic dysfunction and cardiovascular disease (37–39). Radiomics analysis can detect subtle changes in liver morphology, textural heterogeneity, and tissue microstructure imperceptible to the human eye (40, 41). These imaging features may indirectly reflect the metabolic abnormalities and inflammatory processes underlying coronary heart disease development.

Compared with conventional radiomics, DL has the advantage of automatically learning complex hierarchical representations from imaging data without relying on handcrafted feature engineering (42). In our study, the DLR model, which integrated radiomics features and compressed DL features extracted using DenseNet-121, achieved significantly superior predictive performance compared with the radiomics model alone. The DLR model achieved AUCs of 0.895 and 0.854 in the training and test cohorts, respectively. This improvement suggests that DL features provide complementary information beyond traditional radiomics descriptors. While radiomics primarily captures shape, intensity, and texture features from CT images, DL can identify highly abstract imaging patterns and nonlinear relationships that may be associated with CHD risk (43). Similar findings have been reported in previous studies involving lung cancer, gastric cancer, and rectal cancer, where the fusion of radiomics and DL features consistently outperformed either approach alone (44–46).

In our nomogram, the DLR score contributed substantially to the total points, suggesting that imaging-derived deep features offer significant incremental predictive value beyond traditional clinical risk factors. This indicates that the DLR score may capture high-dimensional patterns and abstract phenotypes related to metabolic dysfunction, hepatic microstructural remodeling, and systemic atherosclerotic burden that are not adequately reflected in routine clinical variables. By integrating complementary information from clinical and imaging domains, the proposed nomogram provides a more comprehensive characterization of disease prediction. Moreover, the DeLong test confirmed that the combined model significantly outperformed the other models, supporting the incremental value of clinical and imaging data fusion. In the subgroup analysis, the combined model demonstrated consistent performance across sex subgroups but showed reduced specificity in patients aged ≥60 years. This finding may be partially explained by the application of a uniform classification threshold across different age groups. Since older individuals often have a higher burden of cardiovascular risk factors (47), a fixed threshold may affect model calibration and increase false-positive predictions in this subgroup. Future studies will be further explored with larger cohorts and external validation datasets.

Another notable strength of this study is the adoption of an AI-assisted segmentation strategy. Manual liver segmentation is time-consuming and subject to inter-observer variability, which limits the practical application of radiomics in clinical settings. By employing a deep learning-assisted segmentation framework integrated within ITK-SNAP, we achieved rapid and accurate liver delineation with minimal manual adjustment. This workflow substantially reduced labor costs and improved reproducibility, thereby enhancing the feasibility of large-scale clinical implementation. Recent studies have demonstrated that automatic segmentation facilitates efficient radiomics analysis while maintaining high predictive accuracy (48, 49).

From a clinical perspective, the proposed nomogram represents a non-invasive and easily accessible tool for assessing CHD risk in patients with MAFLD. Given that liver CT examinations are routinely performed in many clinical centers, our fusion model can generate individualized risk estimates for CHD without the need for additional imaging modalities or invasive diagnostic procedures. The satisfactory calibration performance and DCA suggest that the model provides a meaningful net clinical benefit across a broad range of threshold probabilities. Accordingly, this approach holds potential for identifying high-risk individuals who may benefit from early preventive interventions.

## 5 Limitations

Several limitations of this study should be acknowledged. First, this was a retrospective single-center study, which may have introduced selection bias and limited the generalizability of the findings. External multicenter validation is necessary before clinical implementation. Second, although AI-assisted liver segmentation using nnInteractive improved segmentation efficiency and reduced the workload of manual delineation, visual inspection and occasional minor manual corrections were still required in some cases. Further optimization and validation of fully automated segmentation workflows are warranted in future studies. Third, only non-contrast liver CT images were analyzed; incorporating other imaging modalities such as MRI or ultrasound may further improve predictive performance.

## 6 Conclusion

We developed and validated an AI-assisted segmentation-based deep learning radiomics nomogram for identifying individuals at risk of CHD among patients with MAFLD. By integrating clinical parameters, radiomics features, and deep learning features, the proposed DLRC model demonstrated superior predictive performance compared with individual models. This non-invasive and efficient approach may facilitate early CHD risk stratification and support personalized cardiovascular risk management in patients with MAFLD. Future studies involving multicenter external validation, larger prospective cohorts, and integration into clinical decision-support systems are warranted to further evaluate the generalizability, robustness, and clinical utility of the proposed model.

## Data Availability

The raw data supporting the conclusions of this article will be made available by the authors, without undue reservation.
