# Supplementary material for: AI-assisted segmentation-based fusion model integrating deep learning, radiomics, and clinical parameters for identifying coronary heart disease risk in patients with MAFLD
Source: Front Endocrinol (Lausanne). 2026 Aug 7;17:1908048. doi: 10.3389/fendo.2026.1908048 (PMC13504196; doi:10.3389/fendo.2026.1908048)
Supplement: Supplementary file 1 [file Supplementaryfile1.docx]

CT acquisition protocols for different scanners

|  | Somatom Definition AS | Somatom Perspective | GE Optima540 |
| --- | --- | --- | --- |
| Tube voltage (kV) | 120 | 120 | 120 |
| Tube current (mAs) | 40 | 40 | 40 |
| Pitch | 0.758 | 0.758 | 0.758 |
| Collimation (mm) | 0.6×64 | 0.6×64 | 0.6 × 16 |
| Slice thickness (mm) | 1 | 1 | 0.6/1 |
| Matrix | 512 × 512 | 512 × 512 | 512 × 512 |

Radscore = 0.29339622641509433 -0.075312 * original_shape_Maximum2DDiameterColumn -0.062915 * original_shape_Flatness -0.008654 * original_glcm_ClusterShade +0.057932 * original_glszm_ZoneEntropy -0.036771 * original_shape_LeastAxisLength -0.002610 * original_glrlm_RunVariance -0.001886 * original_glszm_ZoneVariance -0.011701 * original_shape_MinorAxisLength -0.070582 * original_glszm_SizeZoneNonUniformityNormalized +0.045568 * original_firstorder_Range -0.028492 * original_shape_SurfaceVolumeRatio

DLRscore = 0.2933962264150943 +0.256068 * DL_1 -0.027412 * original_shape_LeastAxisLength -0.004034 * original_glszm_SmallAreaEmphasis +0.008058 * DL_26 +0.015448 * original_firstorder_Kurtosis -0.031092 * DL_7 -0.063581 * DL_0 -0.030819 * original_shape_Maximum2DDiameterColumn +0.058493 * original_glszm_ZoneEntropy +0.025042 * DL_29 +0.042798 * DL_11 +0.012405 * DL_10 -0.012132 * original_glcm_ClusterShade +0.010995 * DL_18 -0.004361 * original_glszm_GrayLevelNonUniformity

**Supplement Table 1 Performance comparison of ResNet-50 and SimpleViT in the training and test cohorts**

| Signature | ResNet-50 | | | | SimpleViT | | | | Cohort |
| --- | --- | --- | --- | --- | --- | --- | --- | --- | --- |
|  | Accuracy | AUC(95% CI) | Sensitivity | Specificity | Accuracy | AUC(95% CI) | Sensitivity | Specificity |  |
| Clinic | 0.659 | 0.768(0.739 - 0.797) | 0.862 | 0.575 | 0.659 | 0.768(0.739 - 0.797) | 0.862 | 0.575 | train |
| Rad | 0.717 | 0.821(0.795 - 0.848) | 0.836 | 0.668 | 0.717 | 0.821(0.795 - 0.848) | 0.836 | 0.668 | train |
| DLR | 0.850 | 0.900(0.879 - 0.922) | 0.746 | 0.893 | 0.803 | 0.860(0.836 - 0.884) | 0.717 | 0.838 | train |
| Combined | 0.809 | 0.916(0.898 - 0.934) | 0.868 | 0.785 | 0.786 | 0.885(0.865 - 0.906) | 0.852 | 0.758 | train |
| Clinic | 0.648 | 0.751(0.706 - 0.797) | 0.845 | 0.571 | 0.648 | 0.751(0.706 - 0.797) | 0.845 | 0.571 | test |
| Rad | 0.705 | 0.784(0.738 - 0.830) | 0.798 | 0.669 | 0.705 | 0.784(0.738 - 0.830) | 0.798 | 0.669 | test |
| DLR | 0.758 | 0.853(0.813 - 0.892) | 0.822 | 0.733 | 0.752 | 0.799(0.755 - 0.843) | 0.713 | 0.767 | test |
| Combined | 0.745 | 0.872(0.839 - 0.906) | 0.891 | 0.687 | 0.725 | 0.835(0.798 - 0.873) | 0.837 | 0.681 | test |
